# Estivation Strategies of Invasive Snail *Pomacea canaliculata* in Paddy Fields: Roles of Gut Microbiota and Digestive Gland Metabolism

**DOI:** 10.1002/ece3.74091

**Published:** 2026-08-03

**Authors:** Fucheng Yao, Yuchao He, Yingtong Chen, Jiaen Zhang, Zhaoji Shi, Zhong Qin

**Affiliations:** ^1^ Department of Ecology College of Natural Resources and Environment, South China Agricultural University Guangzhou China; ^2^ Guangdong Laboratory for Lingnan Modern Agriculture, Key Laboratory of Agro‐Environment in the Tropics, Ministry of Agriculture and Rural Affairs South China Agricultural University Guangzhou China; ^3^ Guangdong Engineering Technology Research Centre of Modern Eco‐Agriculture and Circular Agriculture South China Agricultural University Guangzhou China; ^4^ School of Environmental Science and Engineering Guangdong University of Technology Guangzhou China

**Keywords:** apple snail, estivation, gut microbiota, invasive mechanism, metabolism

## Abstract

The agricultural pest *Pomacea canaliculata* burrows into soil for estivation after the summer rice harvest, and reemerges to damage crops once late rice irrigation begins, posing a major challenge to crop management. Understanding its strategies during estivation is therefore essential for developing effective control measures. In this study, a 60‐day in situ experiment was conducted during the summer fallow period in a rice field to investigate the survival dynamics, gut microbiota, and metabolic adaptations of estivating snails, aiming to elucidate their oversummering strategies. Our results showed that *P. canaliculata* maintained a high survival rate of up to 92% after 60 days of estivation, even when the maximum soil temperature reached 43°C. Among the 42 medium‐ and long‐chain fatty acids detected in the digestive gland (liver), 39 remained stable, while docosatetraenoic acid increased and caprylic acid and docosapentaenoic acid decreased. During estivation, the gut microbiota underwent pronounced structural shifts, with increased richness, evenness, and diversity. The relative abundance of the dominant phylum *Firmicutes* declined sharply, whereas *Bacteroidota* increased, with community assembly driven mainly by stochastic processes. Short‐chain fatty acid (SCFA)‐producing genera, including anaerobes such as *Paludibacter*, *Bacteroides*, and *Acetobacteroides*, were enriched during estivation. The remodeled gut microbiota may help maintain fatty acid metabolic homeostasis in the digestive gland via the gut‐liver axis. Our findings reveal the adaptive responses of *P. canaliculata* to high‐temperature and drought stress during estivation and highlight the urgency of implementing control measures during the fallow period. Future work should explore agronomic strategies for suppressing *P. canaliculata* populations during this vulnerable stage.

## Graphical abstract

During the summer fallow period, *Pomacea canaliculata* enters estivation under high temperature and drought stress yet maintains a survival rate exceeding 92%. Restructuring of the gut microbiota and metabolic adaptations in the liver, particularly the stability of fatty acid profiles, underpin its strong estivation tolerance.

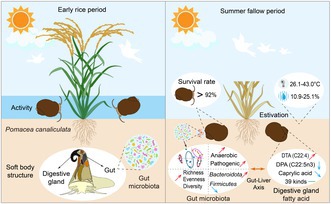

## 1 Introduction

*Pomacea canaliculata* (Gastropoda: Ampullariidae), widely known as the apple snail, is a freshwater gastropod species that originates from the Amazon River Basin of South America (Seuffert and Martín 2013; Yao et al. 2023). Due to its high reproductive rate (Cowie 2002), adaptability (Wada and Matsukura 2011; Yao et al. 2024), and the absence of natural predators in non‐native habitats (Carlsson et al. 2004), *P. canaliculata* has spread to various regions, including Asia, North America, Africa, and Europe (Constantine et al. 2023; Seuffert and Martín 2021). It has become a globally invasive agricultural pest, posing significant threats to both agricultural ecosystems and aquatic biodiversity (Horgan et al. 2021; O'Neil et al. 2023). Notably, *P. canaliculata* was included in the International Union for Conservation of Nature (IUCN) list of the world's 100 worst invasive alien species in the year 2000, where it was the only freshwater snail species represented (Lach et al. 2000). Its voracious appetite for aquatic plants and crops, especially rice seedlings and tillers, has made *P. canaliculata* a notorious pest, causing yield reductions in rice of 10%–90% (Hayes et al. 2008; Litsinger and Estano 1993). Additionally, *P. canaliculata* serves as an intermediate host for various parasites (e.g., *Angiostrongylus cantonensis* ), further exacerbating its detrimental effects on human health and ecosystems (Song et al. 2016).

The invasive snail *P. canaliculata* exhibits remarkable tolerance to various abiotic stresses, including cold (Qin, Wu, et al. 2020; Qin, Yang, et al. 2020), heat (Giraud‐Billoud et al. 2013; Mu et al. 2015), desiccation (Guo et al. 2019; Wada and Matsukura 2011), food shortage (Lach et al. 2000; Yusa et al. 2006), and high salinity (Qin, Wu, et al. 2020; Qin, Yang, et al. 2020; Qin et al. 2022; Yang et al. 2018). This broad spectrum of adaptive capabilities enables it to survive under extreme environmental conditions. Hibernation and estivation are common survival strategies employed by *P. canaliculata* in response to natural seasonal changes, cold snaps and heat waves (Hayes et al. 2015). This ability to enter dormancy allows *P. canaliculata* to persist in agricultural ecosystems, where it poses significant challenges to crop management. After the early rice harvest, the paddy fields gradually dry up, prompting *P. canaliculata* to burrow into the topsoil for estivation (Cowie 2002). Upon irrigation of the late rice fields, these snails reemerge from the soil, posing a renewed threat to rice production. Therefore, understanding the response strategies of *P. canaliculata* during estivation in paddy fields is crucial for developing effective management measures to mitigate its impact on rice production.

Population of *P. canaliculata* is predominantly distributed in tropical and subtropical regions, and research on its estivation has garnered significant attention. During midsummer, *P. canaliculata* encounters challenges such as water scarcity, high temperatures, and food shortage, but the primary factor triggering its estivation is thought to be the drying out of habitats (i.e., the drying of paddy fields during the fallow period), and this process seems to be “passive” (Hayes et al. 2015). Previous laboratory studies have revealed that *P. canaliculata* displays a series of physiological, biochemical, and metabolic responses during the activity‐estivation‐arousal cycle, enabling it to safely wake up from a low metabolic state and resume normal activity within a short period of time (Giraud‐Billoud et al. 2022). *P. canaliculata* adopts a “preparation for oxidative stress” (POS) strategy during estivation. Under laboratory‐simulated estivation, *P. canaliculata* enhances its antioxidant defense system by elevating levels of nonenzymatic antioxidants, such as uric acid and glutathione (GSH), as well as enhancing the activities of key antioxidant enzymes, including superoxide dismutase (SOD) and catalase (CAT), thereby alleviating oxidative stress (Giraud‐Billoud et al. 2011, 2022, 2013). Consistently, proteomic analyses have revealed a marked upregulation of CAT expression following 30 days of estivation (Sun et al. 2013). Additionally, the hemocyanin levels in the blood of *P. canaliculata* increase, while lactate levels remain unchanged during short‐term estivation, indicating that aerobic metabolism remains dominant during this period (Rodriguez et al. 2023). Despite extensive research conducted under controlled laboratory conditions, little is known about the physiological and metabolic dynamics of *P. canaliculata* during estivation in natural habitats.

The gut microbiome plays a significant role in elucidating the biological invasion mechanisms of *P. canaliculata* (Liu et al. 2025). These gut microorganisms of *P. canaliculata* were demonstrated to be essential for stress response (i.e., temperature, pollutant) and food digestion (Bao et al. 2024; Bi et al. 2024; Liu et al. 2018). The gut microbiota of *P. canaliculata* show adaptive adjustments in response to seasonal changes and temperature variability (Li, Qian, Gao, et al. 2022; Li, Qian, Yang, et al. 2022). A close interaction exists between the gut microbiota and hepatic metabolic functions. During hibernation, specific gut microbes facilitate the production of short‐chain fatty acids (SCFAs), particularly acetate, which serves as an important energy source for both intestinal epithelial cells and the host organism (Carey et al. 2013). Acetate produced in the gut can be taken up by the liver and used for fatty acid and cholesterol synthesis or converted into ketone bodies, thereby providing energy to peripheral tissues such as the brain, skeletal muscle, and heart (Heldmaier et al. 2004; Macfarlane and Macfarlane 2003). Furthermore, the gut microbiota of *P. canaliculata* not only acts as a reservoir for pathogenic bacteria and virulence factors but also critically influences the invasion success of this species by affecting the health and survival of the sympatric native snail *Cipangopaludina chinensis* (Liu et al. 2025, 2026). Establishing the connection between the gut microbiota and the liver (the gut‐liver axis) in *P. canaliculata* in response to natural stressors can further elucidate its invasion mechanisms.

In this study, we investigated the survival strategies and physiological adaptations of paddy *P. canaliculata* during estivation, with three specific objectives: (1) to characterize the survival dynamics of snails under natural field conditions throughout the estivation period; (2) to elucidate the dynamic shifts in gut microbiota composition and fatty acid profiles in the digestive gland during estivation; and (3) to explore the potential linkage between gut microbiota alterations and digestive gland metabolic responses, thereby providing insight into the microbiota–host metabolic interplay underlying estivation tolerance in this invasive snail.

## 2 Materials and Methods

### 2.1 Test Snails

*Pomacea canaliculata* snails were maintained in outdoor cement pools (200 cm × 100 cm × 60 cm) at the Ecological Teaching and Research Farm (23°16′ N, 113°36′ E) of South China Agricultural University (SCAU) in Guangzhou, China. The bottom of each pool was covered with topsoil (approximately 30 cm in thickness) sourced from a rice paddy, and the water depth in the habitat was maintained at about 10 cm. The study area experiences a humid subtropical monsoon climate with pronounced seasonal variability. July is the warmest month, while January is the coolest, with a mean annual temperature of 21.5°C. Precipitation is highly seasonal, with over 80% of the annual rainfall (ranging from 1612 to 1909 mm) occurring between April and September.

### 2.2 In Situ Experimental Design

**FIGURE 1:**
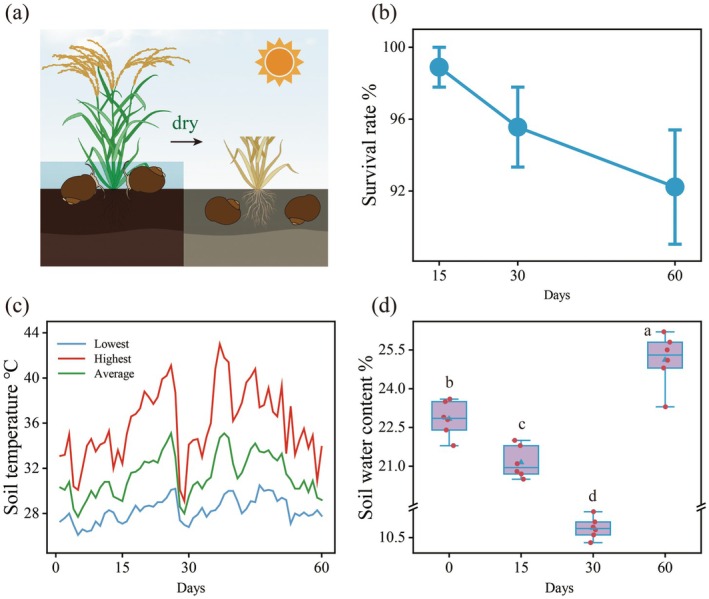
(a) Schematic diagram of *Pomacea canaliculata* during estivation in rice fields. (b) Survival rate of *P. canaliculata* during estivation. (c, d) Soil temperature and soil water content during estivation. *n* = 6. Different lowercase letters indicate significant differences (*p* < 0.05) among estivation periods.

An in situ experiment was performed in a rice paddy at the Ecological Teaching and Research Farm, SCAU, from June 19 to August 18, 2023 (a total of 60 days), to simulate the estivation (summer dormancy) of snails in the surface soil following the rice harvest (Figure 1a). The day before the experiment, the cement tank was drained by opening the drainage outlet to simulate postharvest paddy field drying, prompting the snails to burrow into the soil for dormancy. Female snails with shell heights ranging from 30 to 35 mm that had burrowed into the soil were transferred to the experimental rice field for in situ trials. Adult snails were classified by sex based on the shape of the operculum and shell aperture (Cazzaniga 1990). A white plastic mesh basket (31.5 cm × 23 cm × 10 cm) was filled with soil to a depth of 5 cm, and 15 female snails were evenly placed on the soil surface. Additional soil was then added to cover the snails, filling the basket nearly to the top. The basket was covered with a white plastic mesh bag to prevent wildlife predation and buried flush with the rice field surface. A total of 18 baskets containing 270 female snails were buried. Sampling was conducted at three time points: day 15, day 30, and day 60. At each interval, six baskets were randomly excavated. Snails from each basket were collected into breathable bags and transported to the laboratory for further analysis. Soil samples from each basket were also collected for water content measurement.

### 2.3 Soil Environment Monitoring

Soil temperature was continuously recorded throughout the experiment using an intelligent temperature monitoring system (Beijing CIMC Smart Agriculture Technology Co. Ltd.). The sensor probe was installed at a soil depth of 4–5 cm, and temperature data were automatically transmitted online at hourly intervals (24 readings per day). Based on the 24 hourly temperature readings recorded each day, the daily lowest and highest soil temperatures were determined as the minimum and maximum values among the 24 readings, respectively, while the daily average soil temperature was calculated as the arithmetic mean of the 24 hourly values. The soil water content was quantified using a gravimetric method, with samples dried at 105°C (Yao et al. 2024).

### 2.4 Survival Number of Snails

At each sampling time point, snails were carefully excavated from the soil and transported to the laboratory, where the number of surviving individuals was recorded to calculate survival rates. The survival of each snail was determined based on the absence of putrefaction odor and by gently stimulating the operculum to observe retraction responses.

### 2.5 Digestive Gland and Gut Sample Collection

At each sampling time point, one living snail was randomly selected from each basket. The snail’s shell and body surface was wiped three times with 75% ethanol wipes, and then dissected using sterile scissors to extract its intact soft tissue. The digestive gland of one snail was carefully dissected, transferred into a sterile 2 mL cryovial (Bikeman Biotechnology Co. Ltd., Hunan, China), and immediately snap‐frozen in liquid nitrogen.

Almost simultaneously with the digestive gland sampling, the intestinal tissue of the same snail was quickly separated and placed into another 2 mL sterile cryovial, which was also frozen in liquid nitrogen. All procedures were performed under a sterile workbench. After tissue collection at each time point, samples were transferred to a −80°C freezer for storage until further analysis. At each sampling time point (days 0, 15, 30, and 60), six samples from each of digestive gland and intestinal tissue were collected.

### 2.6 Fatty Acid Determination

A mixed standard solution containing 51 medium‐ and long‐chain fatty acid methyl esters (FAMEs) was prepared and serially diluted to obtain working standard solutions at various concentrations. These solutions were used for Gas Chromatography–Mass Spectrometry (GC–MS) analysis to construct a standard curve for the quantitative analysis.

For sample pretreatment, 50 mg of digestive gland sample was precisely weighed into a 2 mL grinding tube, followed by the addition of a small steel bead and 1 mL of dichloromethane/methanol solution (v/v = 1:1). The mixture was ground using a cryogenic grinder (50 Hz) for 3 min, subjected to low‐temperature ultrasonic treatment for 15 min, and then allowed to stand at −20°C for 15 min. The mixture was centrifuged at 13,000 rcf for 10 min at 4°C, and 500 μL of the supernatant was transferred to a 1.5 mL microcentrifuge tube and dried under a stream of nitrogen. The dried residue was then reconstituted in 0.5 mL of methylation reagent (0.5 mol/L sodium hydroxide in methanol), vortexed for 30 s, sonicated for 10 min at 4°C, and incubated in a 60°C water bath for 30 min. After cooling, 0.5 mL of n‐hexane was added, and the mixture was vortexed for 30 s and centrifuged at 13,000 rcf for 10 min at 4°C. Finally, 100 μL of the upper n‐hexane layer was transferred to a sample vial for GC–MS analysis.

**FIGURE 2:**
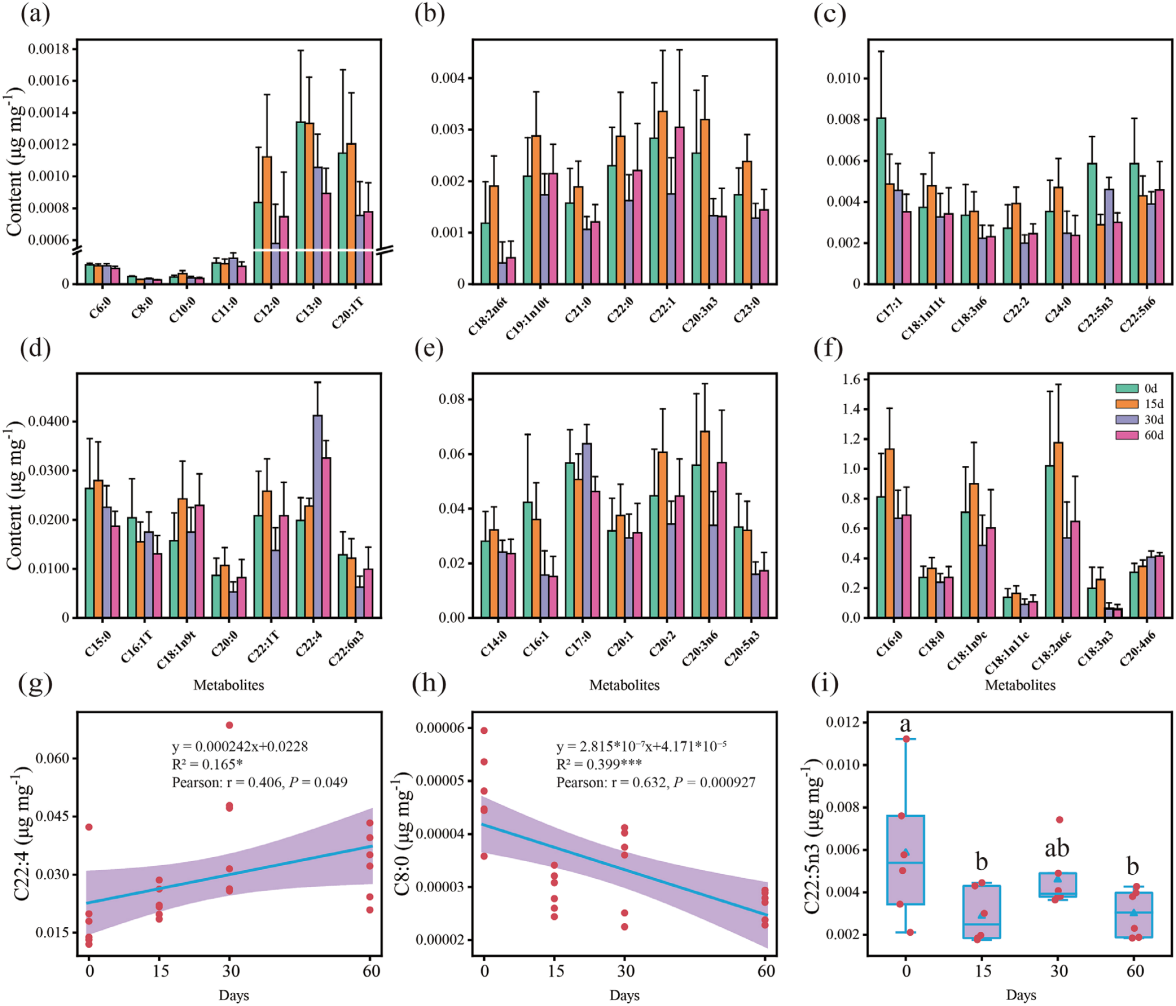
Dynamics of 42 medium‐ and long‐chain fatty acids (a–i) in the digestive gland of *Pomacea canaliculata* during estivation in rice fields. *n* = 6. Different lowercase letters indicate significant differences (*p* < 0.05) among estivation periods. Asterisks indicate significant differences: *p < 0.05, **p < 0.01, ***p < 0.001.

Both working standard solutions and pretreated samples were analyzed by GC–MS under identical conditions, using an 8890B‐7000D GC/MSD system (Agilent Technologies Inc., CA, USA). The concentration of the target compound in the test samples was determined using the standard curve, and the actual content of the target compound was quantified. In total, 42 medium‐ and long‐chain fatty acids (Figure 2a–f) were detected in the digestive gland.

### 2.7 Gut Bacteria Detection

#### 2.7.1 DNA Extraction and 16S rRNA Sequencing

Intestinal microbial DNA was extracted with the FastDNA Kit (MP Biomedicals, CA, USA) in accordance with the supplier's protocol. The quality of the extracted DNA was examined by 1% agarose gel electrophoresis, and DNA purity and concentration were measured using a NanoDrop 2000 spectrophotometer (Thermo Scientific, Wilmington, DE, USA).

The V3–V4 hypervariable region of the bacterial 16S rRNA gene was amplified using primers 338F and 806R. PCR amplicons were purified with the AxyPrep DNA Kit (AXYGEN, USA) and quantified using QuantiFluor‐ST (Promega, USA). High‐throughput sequencing was subsequently conducted on an Illumina NextSeq 2000 platform (Illumina, San Diego, USA) by Majorbio BioPharm Technology Co. Ltd. (Shanghai, China).

#### 2.7.2 Bioinformatic Analysis

Raw sequencing data were analyzed using the QIIME 1.9.1 pipeline. FASTQ files were subjected to demultiplexing and quality filtering, with sequence merging and trimming performed using FLASH v1.2.11 and Trimmomatic, respectively (Bolger et al. 2014; Magoč and Salzberg 2011). Operational taxonomic units (OTUs) were subsequently clustered with UPARSE v7.1 at a 97% sequence similarity cutoff (Edgar 2013). RDP Classifier 2.2 was utilized to assign taxonomy to each 16S rRNA gene sequence by comparing it against the SILVA 138 rRNA database (Bokulich et al. 2018; Quast et al. 2012). Unclassified OTUs at the genus level were taxonomically assigned by BLAST searches against the NCBI database. Alpha‐diversity metrics of the microbial communities were calculated using Mothur v1.30.1 (Schloss et al. 2009), and bacterial phenotypic traits were predicted with BugBase software (Zhang et al. 2019).

### 2.8 Statistical Analysis

Beta diversity of the gut microbial communities was assessed using nonmetric multidimensional scaling (NMDS). Permutational multivariate analysis of variance (PERMANOVA) was conducted with the vegan package based on Bray–Curtis dissimilarity to test differences among groups (Shi et al. 2024). In addition, hierarchical clustering was performed using the UPGMA (Unweighted Pair‐Group Method with Arithmetic Mean) algorithm on the beta‐diversity distance matrix to generate a dendrogram illustrating similarities and differences in gut microbial composition during estivation. The relative contribution of stochastic processes was evaluated by calculating the Phylogenetic Normalized Stochasticity Ratio (pNST) and the Beta Nearest Taxon Index (βNTI) (Dini‐Andreote et al. 2015; Ning et al. 2019). Community assembly processes were further quantified using a phylogenetic bin‐based null model implemented in the iCAMP R package (Ning et al. 2020).

All univariate statistical tests were conducted in IBM SPSS Statistics 20 (IBM Corp., Armonk, NY, USA), with significance set at *p* < 0.05. Pearson correlation and linear regression were used to assess pairwise variable relationships. One‐way ANOVA was applied to compare group means when linear regression yielded non‐significant results. Post hoc multiple comparisons were then performed using the least significant difference (LSD) test for data with homogeneity of variance, or the Games‐Howell test for data with heterogeneity of variance. Graphical visualization of the data was generated using Origin 2024 (OriginLab Corp., Northampton, MA, USA). The schematic diagram in this study was created using Adobe Illustrator 2024 (Adobe Inc., San Jose, CA, USA).

## 3 Results

### 3.1 Environment Conditions and Survival of Snail During Estivation

Soil temperature (Figure 1c) and soil water content (Figure 1d) in the paddy were recorded during the in situ estivation experiment. The average soil temperature during the entire experiment was 31.37°C. During this period, there were 8 days when the soil temperature exceeded 40°C. The maximum soil temperature occurred on day 37 of estivation, reaching 43°C (Figure 1c). The soil water content on day 0, day 15, day 30, and day 60 was 22.83%, 21.15%, 10.87%, and 25.12%, respectively (Figure 1d).

During the estivation period, the survival rate of female snails remained above 90% (Figure 1b). The survival rates on day 15, day 30, and day 60 were 98.89%, 95.56%, and 92.22%, respectively (Figure 1b).

### 3.2 Medium‐ and Long‐Chain Fatty Acids of *P. canaliculata*

We analyzed 51 medium‐ and long‐chain fatty acids in the snail digestive gland, detecting 42 fatty acids (Figure 2). Among these, the levels of 39 fatty acids remained stable during the estivation period, showing no significant differences across the time points. Notably, palmitic acid (C16:0), stearic acid (C18:0), oleic acid (C18:1n9c), vaccenic acid (C18:1n11c), linoleic acid (C18:2n6c), alpha‐linolenic acid (C18:3n3), and arachidonic acid (C20:4n6) were detected at relatively high concentrations in the digestive gland (Figure 2f). However, the content of docosatetraenoic acid (C22:4) exhibited a significant increase throughout the estivation period (Figure 2g), whereas caprylic acid (C8:0) showed a significant decrease over time (Figure 2h). Furthermore, the content of docosapentaenoic acid (C22:5n3) on day 15 and day 60 of estivation was significantly lower compared with that of the pre‐estivation (day 0) level (Figure 2i).

### 3.3 Gut Microbiota of *P. canaliculata*

#### 3.3.1 Beta and Alpha Diversity

**FIGURE 3:**
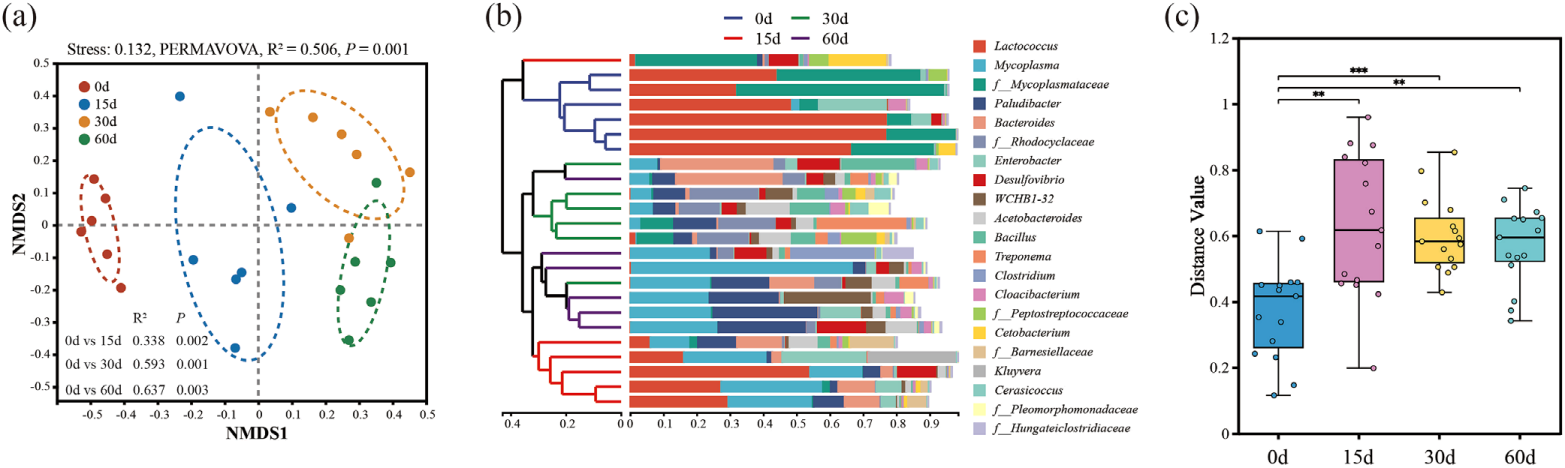
Dynamics of gut microbiota beta diversity in *Pomacea canaliculata* during estivation in rice fields. (a) NMDS and PERMANOVA based on Bray‐Curtis distance. (b) Hierarchical clustering tree at the genus level. (c) Dissimilarity based on Bray‐Curtis distance. *n* = 6. Asterisks indicate significant differences: *p < 0.05, **p < 0.01, ***p < 0.001.

The NMDS plots and PERMANOVA results revealed significant differences in the structure of gut microbiota between day 0 (pre‐estivation) and day 15, day 30, and day 60 of estivation in snails (Figure 3a). Hierarchical clustering analysis further demonstrated that the samples of pre‐estivation were grouped into a separate branch, while the samples from day 15, day 30, and day 60 formed another distinct branch (Figure 3b). Additionally, the beta diversity of gut microbiota during the estivation period (days 15 to 60) was significantly higher compared with that of pre‐estivation (Figure 3c).

**FIGURE 4:**
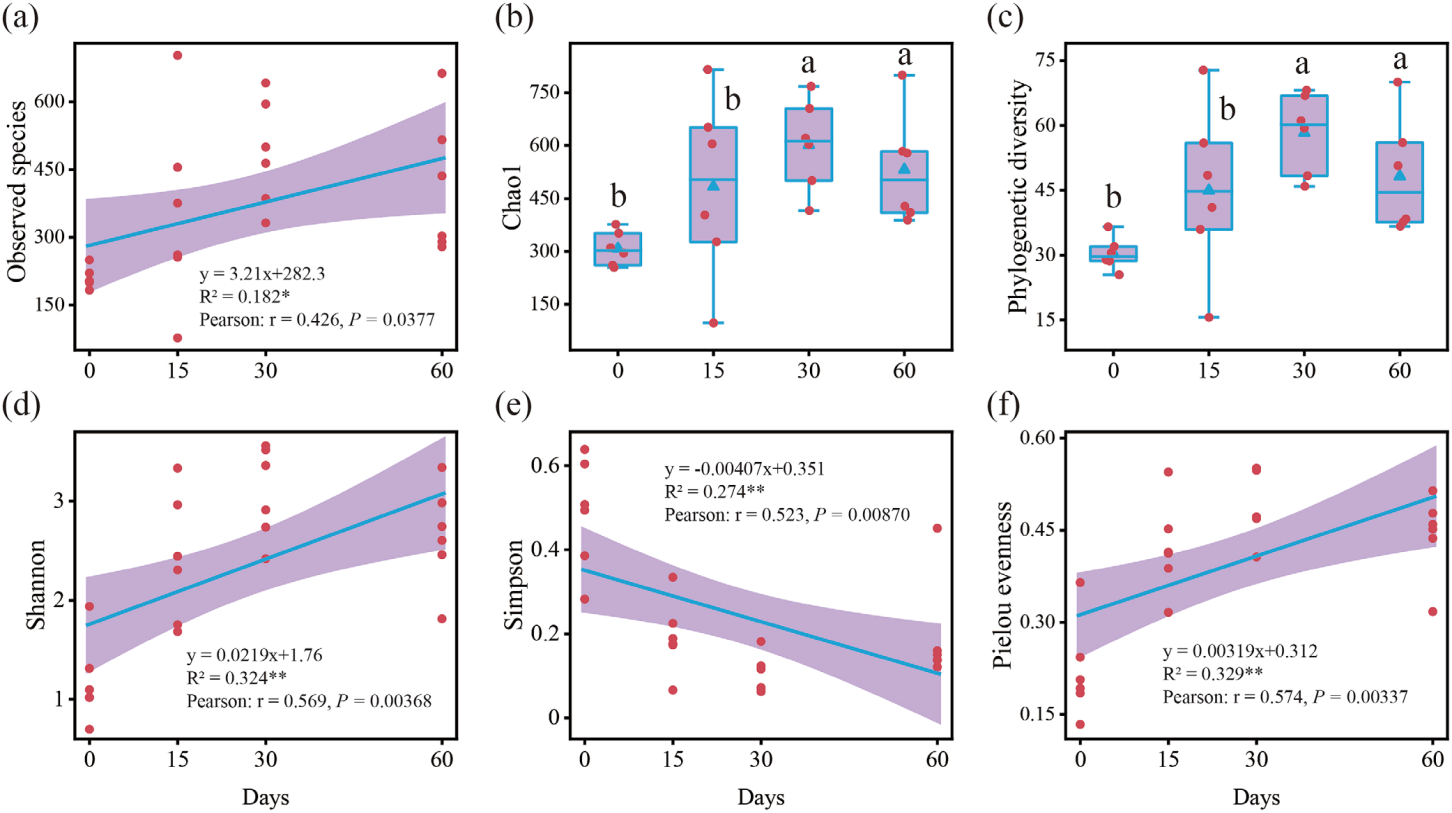
Alpha diversity indices (a–f) of gut microbiota in *Pomacea canaliculata* during estivation in rice fields. *n* = 6. Different lowercase letters indicate significant differences (*p* < 0.05) among estivation periods. Asterisks indicate significant differences: *p < 0.05, **p < 0.01, ***p < 0.001.

We found that the richness (Observed species) of gut microbiota significantly increased with the duration of estivation (Figure 4a). However, the predicted richness (Chao1 index) of gut microbiota on day 15 of estivation did not show a significant difference compared with that of pre‐estivation (Figure 4b). The phylogenetic diversity of gut microbiota on day 30 and day 60 of estivation was significantly higher than that before estivation (Figure 4c). In addition, the diversity (Shannon and Simpson indices) and evenness (Pielou evenness) of gut microbiota significantly increased with the duration of estivation (Figure 4d–f).

#### 3.3.2 Community Composition

**FIGURE 5:**
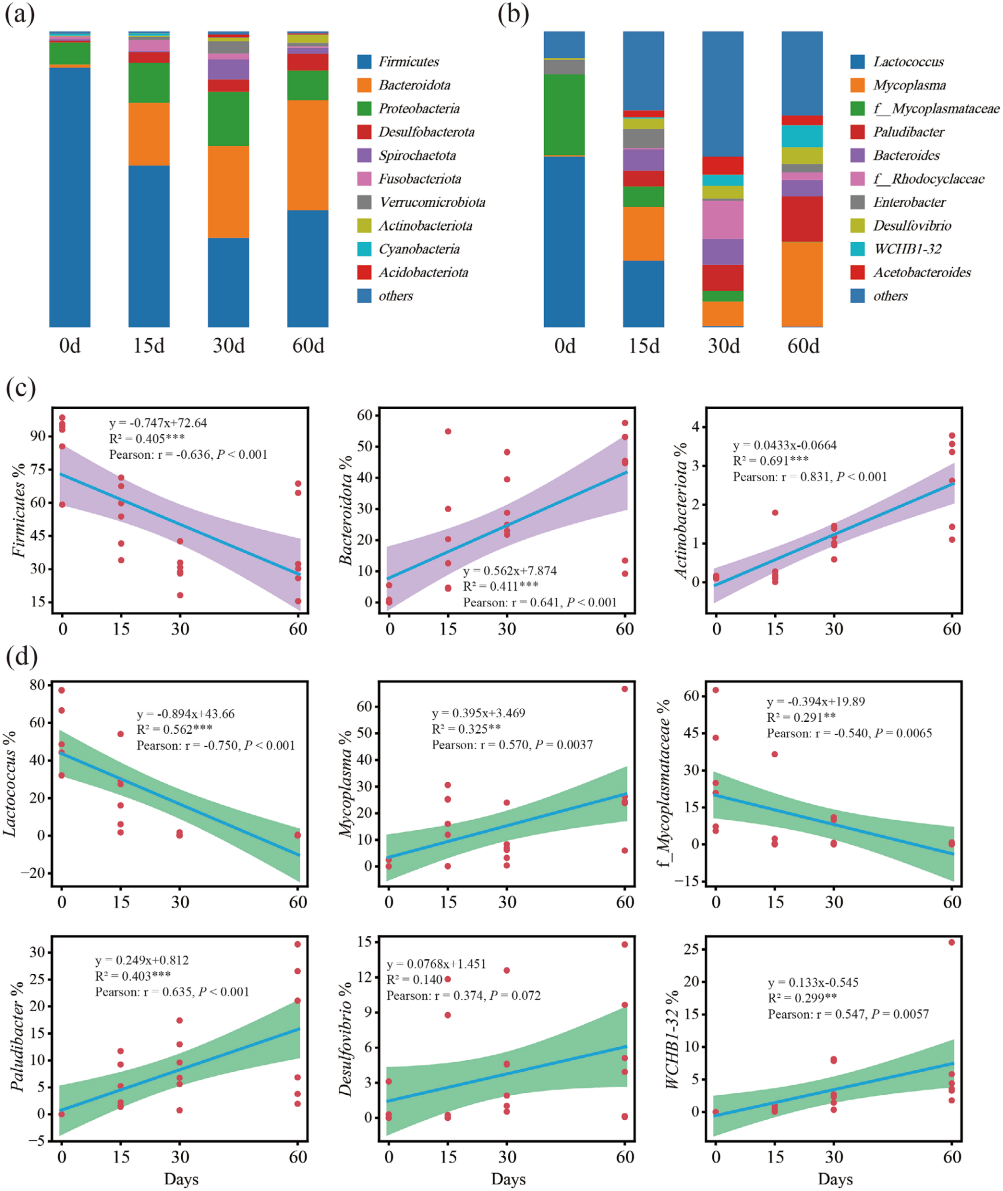
Community composition of gut microbiota in *Pomacea canaliculata* during estivation in rice fields. (a) Community composition at the phylum level. (b) Community composition at the genus level. (c) Dynamics of dominant phyla. (d) Dynamics of dominant genera. *n* = 6. Asterisks indicate significant differences: *p < 0.05, **p < 0.01, ***p < 0.001.

The gut microbial composition of snails differed markedly before and during estivation at both the phylum (Figure 5a) and genus (Figure 5b) levels. Before estivation, the dominant phylum in the gut microbiota was *Firmicutes*. During estivation, the dominant phyla changed to *Firmicutes* and *Bacteroidota* (Figure 5a). Similarly, after entering estivation, the dominant genera in the snail gut microbiota gradually changed from *Lactococcus* to *Mycoplasma* (Figure 5b).

At the phylum level (Figure 5c), the proportion of *Firmicutes* in the snail gut microbiota declined significantly over the course of estivation, decreasing from 87.71% before estivation to 39.51% on day 60. In contrast, the relative abundances of *Bacteroidota* and *Actinobacteriota* showed significant increases with the duration of estivation. Specifically, *Bacteroidota* increased from 1.10% (day 0) to 37.24% (day 60), while *Actinobacteriota* rose from 0.13% (day 0) to 2.64% (60th day).

At the genus level (Figure 5d), the proportion of *Lactococcus* significantly decreased over time during estivation, from 57.69% (day 0) to 0.15% (day 60). However, the relative abundances of *Mycoplasma*, *Paludibacter*, and *WCHB1‐32* significantly increased with the duration of estivation. Specifically, the proportion of these microorganisms increased from 0.43%, 0.003%, and 0% (pre‐estivation) to 28.53%, 15.29%, and 7.48% (day 60), respectively.

#### 3.3.3 Phenotypes and Assembly

**FIGURE 6:**
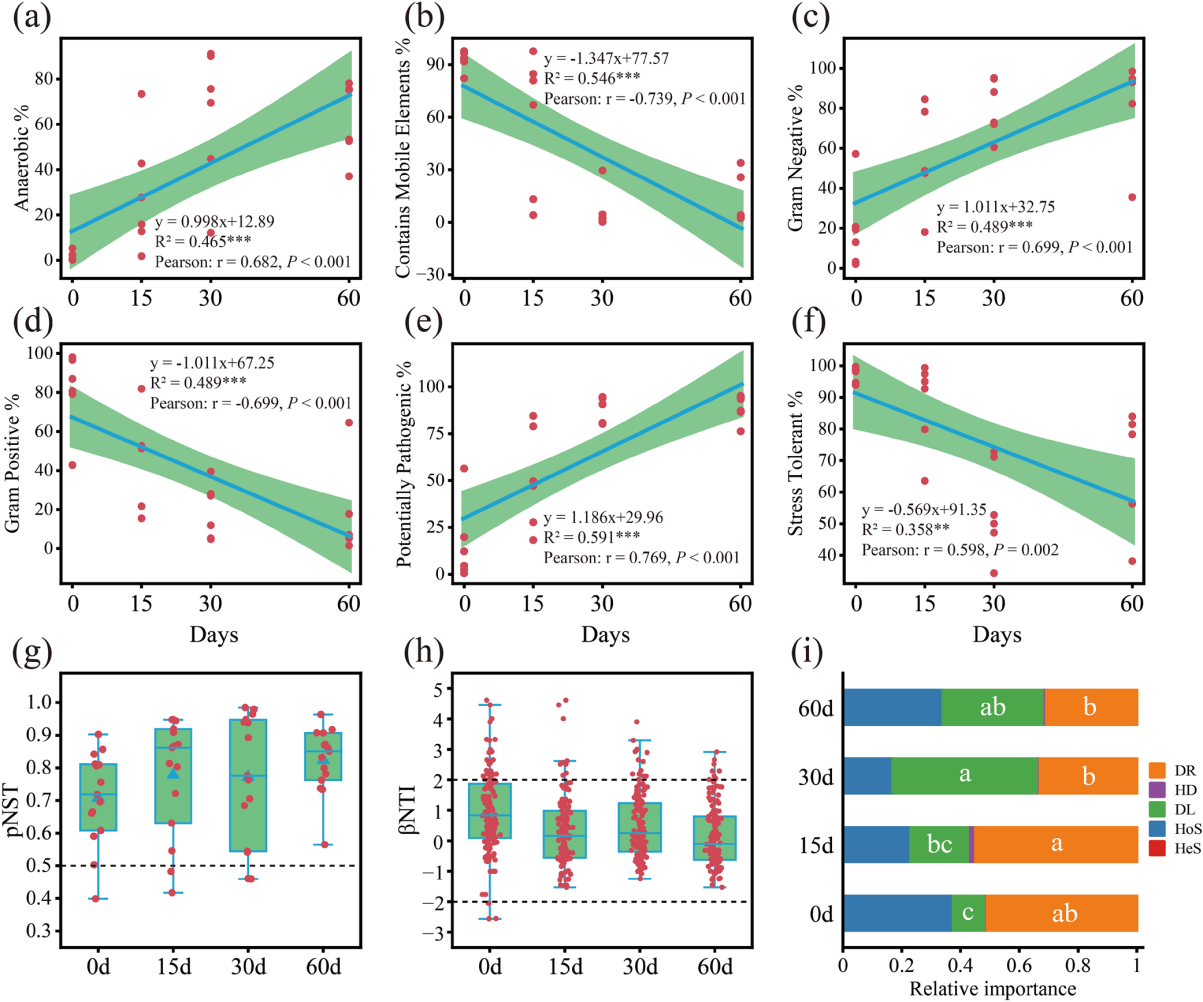
Bacterial phenotypes and community assembly of gut microbiota in *Pomacea canaliculata* during estivation in rice fields. (a–f) Bacterial phenotypes based on BugBase. (g) Phylogenetic Normalized Stochasticity Ratio (pNST). (h) Beta Nearest Taxon Index (βNTI). (i) Ecological process of community assembly based on iCAMP. *n* = 6. DL, dispersal limitation; DR, drift (and others); HD, homogenizing dispersal; HeS, heterogeneous selection; HoS, homogeneous selection. Different lowercase letters indicate significant differences (*p* < 0.05) among estivation periods. Asterisks indicate significant differences: *p < 0.05, **p < 0.01, ***p < 0.001.

Phenotypic predictions of the gut microbiota generated using the BugBase database revealed significant shifts in microbial phenotypes following the onset of estivation (Figure 6). The proportions of microorganisms exhibiting anaerobic (Figure 6a), Gram‐negative (Figure 6c), and potentially pathogenic (Figure 6e) phenotypes showed a continuous increase during estivation, rising from 1.52%, 19.28%, and 16.03% (day 0) to 61.96%, 83.09%, and 88.83% (day 60), respectively. In contrast, the relative abundances of microorganisms that contain mobile elements (Figure 6b), Gram‐positive (Figure 6d), and stress tolerant (Figure 6f) phenotypes exhibited a continuous decline during estivation, decreasing from 92.39%, 80.72%, and 97.28% (day 0) to 11.85%, 16.91%, and 65.74% (day 60), respectively.

The results from pNST, βNTI, and iCAMP analyses collectively demonstrated that the assembly of the snail gut microbiota was predominantly governed by stochastic processes, irrespective of estivation status (Figure 6g–i). During estivation, the pNST values increased, indicating a higher degree of stochasticity in the gut microbiota during this period (Figure 6g). Both βNTI and iCAMP analyses further supported a predominance of stochastic processes in gut microbiota assembly during estivation (Figure 6h–i). |βNTI| ≥ 2 was interpreted as evidence of deterministic processes, whereas |βNTI| < 2 indicated stochastic processes. Consistent with this, iCAMP results showed that deterministic mechanisms comprised homogeneous and heterogeneous selection, while stochastic components included dispersal limitation, homogenizing dispersal, and ecological drift. Specifically, the iCAMP analysis revealed that, compared with the pre‐estivation period, the relative importance of dispersal limitation to the gut microbiota assembly significantly increased on day 30 and day 60 of estivation. In contrast, the relative contribution of drift to the gut microbiota assembly significantly decreased on day 30 and day 60 of estivation compared with that of day 15 (Figure 6i).

### 3.4 Association of Digestive Gland Metabolism With Gut Microbiota

**FIGURE 7:**
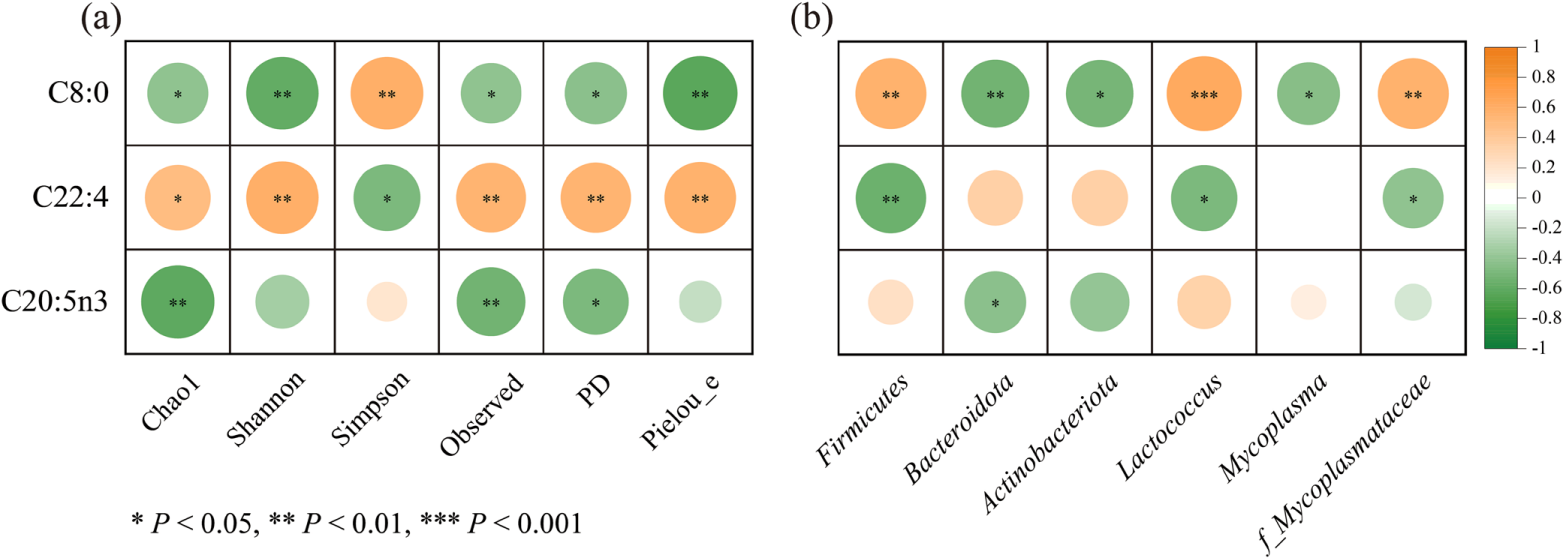
Relationship between digestive gland metabolism and gut microbiota in *Pomacea canaliculata* during estivation in rice fields. (a) Relationships between the three metabolites of digestive gland and alpha diversity indices of gut microbiota. (b) Relationships between the three metabolites of the digestive gland and the dominant phyla and genera of gut microbiota. PD, phylogenetic diversity; Pielou_e, Pielou evenness. *n* = 6. Asterisks indicate significant differences: *p < 0.05, **p < 0.01, ***p < 0.001.

The content of caprylic acid (C8:0) showed a significant negative correlation with the richness, diversity, phylogenetic diversity, and evenness of gut microbiota. In contrast, the content of docosatetraenoic acid (C22:4) exhibited a significant positive correlation with the richness, diversity, phylogenetic diversity, and evenness of gut microbiota. Additionally, the content of docosapentaenoic acid (C22:5n3) displayed a significant negative correlation with both the richness and phylogenetic diversity of the gut microbiota (Figure 7a).

The content of caprylic acid exhibited a significant positive correlation with the abundance of *Firmicutes* and *Lactococcus*. Conversely, the content of caprylic acid showed a significant negative correlation with the proportions of *Bacteroidota*, *Actinobacteriota*, and *Mycoplasma*. The content of docosatetraenoic acid displayed a significant negative correlation with the abundance of *Firmicutes* and *Lactococcus*. Additionally, the content of docosapentaenoic acid demonstrated a significant negative correlation with the proportion of *Bacteroidota* (Figure 7b).

## 4 Discussion

### 4.1 Excellent Adaptability of Snails During Estivation

**FIGURE 8:**
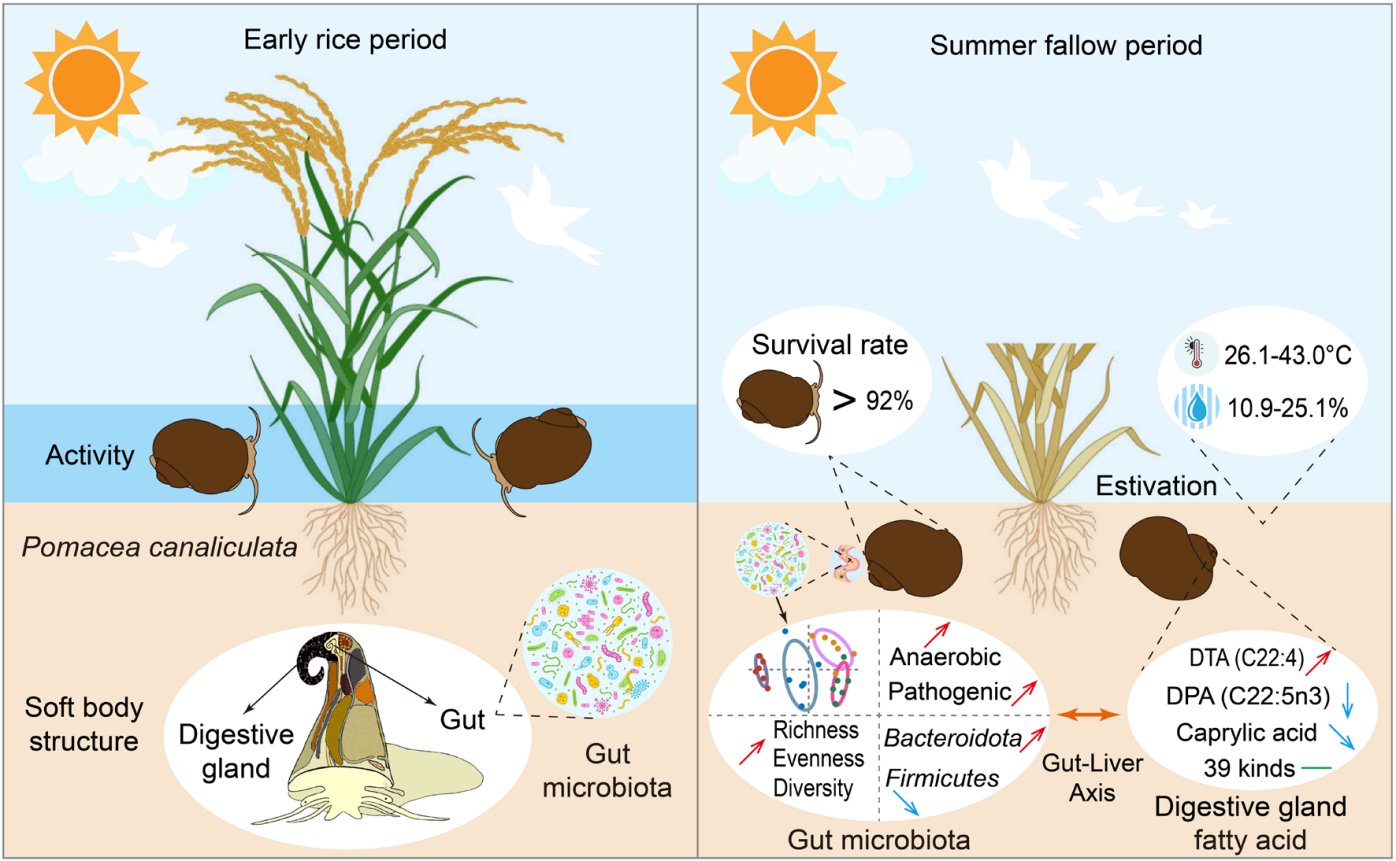
Schematic diagrams showing changes in survival, gut microbiota, and digestive gland metabolism of *Pomacea canaliculata* during estivation in the fallow period of summer rice fields, based on the obtained results. DPA, docosapentaenoic acid; DTA, docosatetraenoic acid. “↗” and “↘” represent positive and negative correlation with time, respectively; “↓” represents a decrease; and “—” represents no significant difference.

During the 60‐day period of the in situ estivation experiment, the highest soil temperature reached 43°C, with the temperature exceeding 40°C on 8 days (Figure 1c). Meanwhile, the minimum soil water content was as low as 10.87% (Figure 1d). This indicates that during estivation, *P. canaliculata* snails faced severe challenges such as fasting, high temperatures, and drought. However, they demonstrated remarkably excellent viability during the dry fallow period after early rice harvest (Figure 1b). In this study, the survival rate of female snails remained as high as 92.22% after 60 days of estivation in the rice fields (Figure 1b; Figure 8). In a laboratory estivation simulation experiment (where *P. canaliculata* was exposed to air at 24°C–26°C), the survival rate of snails after 60 days of estivation in air was approximately 70%, decreasing to 46% after 75 days (Giraud‐Billoud et al. 2011). After 45 days of estivation in air, the snails experienced a weight loss of up to 51%; however, their weight recovered to over 90% after 2 days of rehydration and awakening (Giraud‐Billoud et al. 2011). After 10 months of estivation in air at 20°C–21°C with 60% relative humidity, 50% of *Pomacea maculata* snails still survived (Mueck et al. 2018). Some studies have reported that *P. canaliculata* can survive in soil for over 3 months (Schnorbach 1995) and even more than 7 months under completely dry soil conditions (Mochida 1991). Under laboratory conditions, *P. canaliculata* burrows into the soil as water levels recede gradually at temperatures of 20°C–26°C. Under these conditions, snails can survive for up to 11 months in completely dry soil, and a few individuals can survive for as long as 29 months under moist conditions (Yusa et al. 2006). Another study reported that, after 60 days of estivation in dry soil, the survival rates of female and male *P. canaliculata* were 90% and 72.5%, respectively, while after 120 days, the survival rates of female and male snails were 80% and 53.33%, respectively (Zhang et al. 2023). Similarly, our previous experiment demonstrated that female snails achieved a survival rate as high as 87.5% after 120 days of hibernation in drought‐affected paddy fields during winter (Yao et al. 2024). Such remarkable survival capacity during estivation is not unique to *P. canaliculata* ; similar adaptive strategies have also been observed in other animals facing seasonal drought. For instance, the African lungfish ( *Protopterus annectens* ) burrows deep underground to estivate and secretes mucus during the dry season to form a cocoon composed of mucus and soil around its body, thereby preventing water loss and bacterial infection while awaiting the return of the rainy season (Casadei and Salinas 2023; Heimroth et al. 2021). Overall, adult female snails exhibit a post‐estivation survival rate of up to 92%, which facilitates their rapid reproduction and population recovery after the late rice fields are irrigated, thereby posing a renewed threat to the rice crops.

### 4.2 Dynamics of Fatty Acids in Digestive Gland of Snails During Estivation

The digestive gland of *P. canaliculata* , a pivotal organ responsible for nutrient processing and storage as well as involved in various physiological processes, plays a critical role during estivation (Cowie 2002; Giraud‐Billoud et al. 2022; Hayes et al. 2015). The digestive gland of *P. canaliculata* can tolerate the oxidative burst during estivation through a robust defense mechanism based on enzymatic and non‐enzymatic antioxidant systems (Giraud‐Billoud et al. 2011, 2022, 2013). However, the metabolic dynamics within the digestive gland of *P. canaliculata* during estivation have received little attention. In this study, we investigated the dynamic changes of medium‐ and long‐chain fatty acids (42 kinds were detected) in the digestive gland of *P. canaliculata* during estivation (Figure 2). Notably, 39 of these fatty acids exhibited no significant changes throughout the estivation period (Figure 8). Medium‐ and long‐chain fatty acids of animals play multifaceted and critical roles in energy metabolism, membrane structure maintenance, signal transduction, and immune regulation (Mistry et al. 2021; Nakamura et al. 2014; Schönfeld and Wojtczak 2016). Our results showed that palmitic acid (C16:0), oleic acid (C18:1n9c), and linoleic acid (C18:2n6c) were the predominant long‐chain fatty acids in the digestive gland of *P. canaliculata* (Figures 2f and 8), which is consistent with previous findings (Lavarías et al. 2023). During estivation, the docosatetraenoic acid (C22:4, DTA, adrenic acid) content in the digestive gland of *P. canaliculata* increased progressively with duration (Figure 2g). DTA is a polyunsaturated fatty acid (PUFA) that serves as an important component of cell membrane phospholipids, playing a role in maintaining membrane fluidity, signal transduction, and inflammation regulation (Kopf et al. 2010; Wang et al. 2025). In contrast, the contents of caprylic acid (C8:0) and docosapentaenoic acid (C22:5n3, DPA) in the digestive gland significantly decreased (Figure 2h,i). Caprylic acid is readily absorbed and transported by the liver, where it rapidly enters mitochondria for β‐oxidation, serving as a key fatty acid for rapid energy release (Bach and Babayan 1982; Lemarié et al. 2016). During estivation, snails entered a fasting state, and the caprylic acid stored in the digestive gland may be preferentially mobilized to sustain the organism's energy metabolism. The decline in DPA levels may be attributed to its conversion into other functionally important lipid mediators, such as resolvins, which may play key roles in regulating inflammatory responses during the low metabolic state of estivation (Sánchez‐García et al. 2023).

### 4.3 Dynamics of Snail Gut Microbiota During Estivation

During hibernation, the gut microbiota exhibits adaptive shifts in both composition and function, enabling the host to better tolerate low environmental temperatures and extended periods of fasting (Cao et al. 2024; Regan et al. 2022; Yao et al. 2025). For instance, in ground squirrels, urease‐producing gut microbes increase over the hibernation season, coinciding with elevated host urea transporter expression in intestinal tissues and greater incorporation of recycled nitrogen into proteins (Regan et al. 2022). However, compared with studies on gut microbiota during hibernation, the gut microbial dynamics during estivation remain poorly understood. In this study, we found that the structure of gut microbiota in *P. canaliculata* changed significantly during estivation (Figures 3 and 8), consistent with findings in sea cucumbers ( *Apostichopus japonicus* ) (Kang et al. 2023; Zhao et al. 2024). In contrast, pronounced shifts in the gut microbial community structure of *P. canaliculata* during hibernation were detected only after 60 days (Yao et al. 2025), suggesting a more rapid microbial response in the snail’s gut during estivation. Moreover, the diversity, richness, evenness, and phylogenetic diversity of the gut microbiota in *P. canaliculata* increased significantly during estivation (Figures 4 and 8). In contrast, gut microbial richness remained relatively stable during hibernation in this species (Yao et al. 2025). The findings suggest that more extensive interactions between the gut microbiota and external microbes may occur during estivation. A more diverse gut microbial community is generally considered to confer greater resilience against environmental fluctuations and contributes to the overall stability of the gut ecosystem (Fassarella et al. 2021).

During estivation, the gut microbial composition of *P. canaliculata* underwent pronounced changes. Prior to estivation, *Firmicutes* strongly dominated the gut microbiota, representing 87.7% of the total relative abundance. A high proportion of this phylum is typically associated with herbivorous diets (Sun et al. 2023). Although *P. canaliculata* is an omnivorous species, it shows a dietary preference for crops, phytoplankton, and aquatic plants (Yao et al. 2023). *Firmicutes* play a key role in the degradation of dietary fiber, likely facilitating the digestion of plant‐derived food sources in *P. canaliculata* (Sun et al. 2023). During estivation, *P. canaliculata* enters a fasting state. As residual food is digested, the gut environment shifts, leading to adaptive changes in the microbial community. By day 60 of estivation, the relative abundance of *Firmicutes* had declined to 39.5%, primarily due to a dramatic reduction in the genus *Lactococcus*—from 57.7% before estivation to just 0.2%. *Lactococcus* is mainly involved in the metabolism of dietary carbohydrates (Buron‐Moles et al. 2019), and its decline likely reflects the absence of exogenous nutrients during fasting. In contrast, the relative abundance of *Bacteroidota* increased substantially, from 1.1% before estivation to 37.2% by day 60. Members of this phylum can metabolize host‐derived polysaccharides and utilize endogenous proteins and lipids released from the intestinal epithelium (Sonnenburg et al. 2005; Wu et al. 2011). These capabilities likely allow them to persist and proliferate under host fasting conditions, supporting host energy homeostasis during estivation.

During estivation, the relative abundance of anaerobic bacteria in the gut of *P. canaliculata* increased markedly, rising from 1.5% at day 0 to 62% by day 60 (Figures 6a and 8). As estivation slows the metabolic rate of the snails and reduces gut motility (Rodriguez et al. 2023), an anaerobic environment may develop in the gut of snails, thereby promoting the growth of anaerobic bacteria. Some of these microbes ferment complex substrates, such as polysaccharides and proteins, generating short‐chain fatty acids (SCFAs) that supply energy to the host during dormancy and contribute to the protection of the intestinal mucosa (Sommer et al. 2016; Sonoyama et al. 2009). Notably, the proportion of potential pathogens in the snail gut also increased significantly, from 16% on day 0 to 88.8% on day 60 of estivation (Figure 6e). This suggests that gut dysbiosis occurs during estivation, which may account for the decreased gut immunity. The results could be supported by the decline in the proportion of stress‐tolerant bacteria (Figure 6f). Additionally, the proportion of Gram‐negative bacteria increased sharply from 19.3% on day 0 to 83.1% on day 60 (Figure 6c). Gut dysbiosis was demonstrated to be closely associated with an increase in Gram‐negative bacteria (Salguero et al. 2019; Violi et al. 2023). In fecal microbiota from patients with ulcerative colitis, the proportion of Gram‐negative bacteria is significantly higher than that in healthy individuals (Vigsnæs et al. 2012).

A key objective in ecology is to elucidate the mechanisms of community assembly that shape community composition, diversity, and function (Zhou and Ning 2017). In this study, the assembly of gut microbiota in *P. canaliculata* before estivation (summer) was primarily driven by stochastic processes (Figure 6g–i). In contrast, the assembly of gut microbiota in *P. canaliculata* before hibernation (winter) was dominated by deterministic processes (Yao et al. 2025). Previous studies have shown that the gut microbiota diversity of *P. canaliculata* is lower in winter than in summer (Li, Qian, Gao, et al. 2022; Li, Qian, Yang, et al. 2022), with higher determinism in community assembly correlating with reduced microbial diversity (Shi et al. 2024). Regardless of whether it was during estivation or hibernation, the assembly of gut microbiota in *P. canaliculata* became more stochastic (Figure 6g). Niche‐based theory suggests that deterministic processes, such as environmental filtering (e.g., pH, temperature, humidity, salinity) and biotic interactions (e.g., competition, facilitation, mutualisms, predation), primarily shape community composition (Chave 2004; Chesson 2000). In contrast, neutral theory assumes ecological equivalence among species and attributes community dynamics primarily to stochastic processes, including birth–death events, speciation and extinction, and dispersal (Chave 2004; Hubbell 2001). The compositional shifts and increased diversity of the gut microbiota in *P. canaliculata* during estivation were linked to a predominantly stochastic mode of community assembly.

### 4.4 Association of Digestive Gland Metabolism With Gut Microbiota During Estivation

The gut microbiota closely interacts with the liver via the gut–liver axis to jointly regulate host metabolism and immune functions (Kindt et al. 2018; Schoeler et al. 2023; Wu et al. 2024). Specifically, some gut microorganisms ferment dietary fibers, generating short‐chain fatty acids (SCFAs) such as acetate, propionate, and butyrate (Hays et al. 2024; Mukhopadhya and Louis 2025). These metabolites not only serve as energy sources for intestinal epithelial cells but also influence hepatic lipid metabolism (Ma et al. 2022). For instance, acetate derived from the gut can be utilized by the liver for fatty acid and cholesterol synthesis (Macfarlane and Macfarlane 2003). During estivation, the suspension of food intake induces drastic alterations in the intestinal environment, driving adaptive remodeling of the gut microbiota in *P. canaliculata* . We observed a marked decline in the relative abundance of *Lactococcus* (belonging to *Firmicutes*), which nearly disappeared by day 60 of estivation (Figure 5d). Moreover, the abundance of *Lactococcus* was positively correlated with caprylic acid levels in the digestive gland (Figure 7). Given the interruption of external nutrient intake, *P. canaliculata* may preferentially mobilize stored medium‐chain fatty acids (such as caprylic acid) in the digestive gland (liver) for rapid energy release, thereby maintaining metabolic homeostasis. In addition, SCFA‐producing genera, including *Bacteroides* and *Acetobacteroides*, showed a substantial increase during estivation (Figure 5b). Meanwhile, anaerobic bacteria such as *Paludibacter* (belonging to *Bacteroidota*) and *WCHB1‐32* (belonging to *Spirochaetota*) proliferated significantly during estivation (Figure 5d). *Paludibacter* can ferment complex polysaccharides to generate SCFAs such as acetate and propionate (Gronow et al. 2011; Ueki et al. 2006). These bacteria may degrade residual organic substrates in the gut or mucus (rich in polysaccharides) secreted by the host to produce SCFAs. These fermentation products may then be transported to the digestive gland, serving as supplementary substrates for fatty acid maintenance, membrane fluidity, signal transduction, and inflammation regulation. Overall, during estivation, the gut microbiota of *P. canaliculata* undergoes structural reorganization in response to the metabolic stress of food deprivation. This remodeling may contribute to coordinated energy metabolism and immune regulation via the gut–liver axis, thereby supporting physiological homeostasis during dormancy.

## 5 Conclusion

This 60‐day in situ experiment investigated the survival dynamics, gut microbiota, and metabolic adaptations of *Pomacea canaliculata* during natural summer estivation in paddy fields. *P. canaliculata* exhibited excellent adaptability during natural summer estivation under high‐temperature and drought conditions in paddy soils, maintaining a survival rate of 92% after 60 days. A total of 42 medium‐ and long‐chain fatty acids were detected in its digestive gland, among which 39 fatty acids remained stable throughout the estivation period. Notably, the level of docosatetraenoic acid increased, whereas caprylic acid and docosapentaenoic acid levels declined. Significant shifts in the gut microbial community structure were observed starting from day 15 of estivation. Microbial richness, evenness, and diversity all increased significantly during estivation. The relative abundance of the dominant phylum *Firmicutes* declined substantially, while *Bacteroidota* increased. Additionally, the abundance of anaerobic, potentially pathogenic, and Gram‐negative bacteria increased over time, whereas stress tolerant bacteria declined. These structural and functional changes in the gut microbiota were predominantly governed by stochastic assembly processes. Moreover, SCFA‐producing genera, including *Paludibacter*, *Bacteroides*, and *Acetobacteroides*, were enriched during estivation. These adaptive microbial shifts may contribute to fatty acid metabolic homeostasis in the digestive gland. These findings emphasize the exceptional physiological tolerance and adaptability of *P. canaliculata* during summer estivation in the field.

## Author Contributions

**Fucheng Yao:** conceptualization (equal), data curation (equal), formal analysis (equal), investigation (equal), methodology (equal), writing – original draft (equal), writing – review and editing (equal). **Yuchao He:** investigation (equal). **Yingtong Chen:** investigation (equal). **Jiaen Zhang:** conceptualization (equal), funding acquisition (equal), methodology (equal), project administration (equal), supervision (equal), writing – review and editing (equal). **Zhaoji Shi:** investigation (equal). **Zhong Qin:** conceptualization (equal), funding acquisition (equal), methodology (equal), project administration (equal), supervision (equal), writing – review and editing (equal).

## Funding

This study was funded by the National Natural Science Foundation of China (41871034, 31870525), the Alien Invasive Species Survey Project of the Ministry of Agriculture and Rural Affairs (H20240742), the Guangdong Modern Agricultural Technology Innovation Team Construction Project (2022 KJ134, 2023 KJ134, 2023 KJ105), the Open Project of Guangdong Provincial Key Laboratory of Agricultural Artificial Intelligence (GDKL‐AAI‐202), the Laboratory of Lingnan Modern Agriculture Project (NT2021010), and the Guangdong Science and Technology Program (2019B030301007).

## Conflicts of Interest

The authors declare no conflicts of interest.

## Data Availability

The raw data is available in SRA (https://www.ncbi.nlm.nih.gov/sra) under BioProject numbers PRJNA1418499.
